# Age, period, and cohort analysis of regular dental care behavior and edentulism: A marginal approach

**DOI:** 10.1186/1472-6831-11-9

**Published:** 2011-03-17

**Authors:** Kar-Yan Li, May Chun Mei Wong, Kwok-Fai Lam, Eli Schwarz

**Affiliations:** 1 Dental Public Health, Faculty of Dentistry, The University of Hong Kong, Prince Philip Dental Hospital, 34 Hospital Road, Hong Kong SAR, China; 2 Department of Statistics and Actuarial Science, Faculty of Science, The University of Hong Kong, Pokfulam, Hong Kong SAR, China; 3 Faculty of Dentistry, The University of Sydney, Sydney, NSW 2006, Australia

## Abstract

**Background:**

To analyze the regular dental care behavior and prevalence of edentulism in adult Danes, reported in sequential cross-sectional oral health surveys by the application of a marginal approach to consider the possible clustering effect of birth cohorts.

**Methods:**

Data from four sequential cross-sectional surveys of non-institutionalized Danes conducted from 1975-2005 comprising 4330 respondents aged 15+ years in 9 birth cohorts were analyzed. The key study variables were seeking dental care on an annual basis (ADC) and edentulism. For the analysis of ADC, survey year, age, gender, socio-economic status (SES) group, denture-wearing, and school dental care (SDC) during childhood were considered. For the analysis of edentulism, only respondents aged 35+ years were included. Survey year, age, gender, SES group, ADC, and SDC during childhood were considered as the independent factors. To take into account the clustering effect of birth cohorts, marginal logistic regressions with an independent correlation structure in generalized estimating equations (GEE) were carried out, with PROC GENMOD in SAS software.

**Results:**

The overall proportion of people seeking ADC increased from 58.8% in 1975 to 86.7% in 2005, while for respondents aged 35 years or older, the overall prevalence of edentulism (35+ years) decreased from 36.4% in 1975 to 5.0% in 2005. Females, respondents in the higher SES group, in more recent survey years, with no denture, and receiving SDC in all grades during childhood were associated with higher probability of seeking ADC regularly (*P *< 0.05). The interaction of SDC and age (*P *< 0.0001) was significant. The probabilities of seeking ADC were even higher among subjects with SDC in all grades and aged 45 years or older. Females, older age group, respondents in earlier survey years, not seeking ADC, lower SES group, and not receiving SDC in all grades were associated with higher probability of being edentulous (*P *< 0.05).

**Conclusions:**

With the use of GEE, the potential clustering effect of birth cohorts in sequential cross-sectional oral health survey data could be appropriately considered. The success of Danish dental health policy was demonstrated by a continued increase of regular dental visiting habits and tooth retention in adults because school dental care was provided to Danes in their childhood.

## Background

### Age, Period, and Cohort effects

The need for population-based oral epidemiological studies has long been advocated to determine the oral health or behavior of a population, set targets for the future, and to plan oral health services appropriately [1]. Furthermore, it is important to study changes in oral health (oral health trends) if the oral health care delivery system is to be adapted to best serve the population's needs [2]. Age, Period, and Cohort (APC) analysis has been used by epidemiologists to identify and interpret temporal changes in health characteristics or behaviors in medical and dental research [3-10]. The APC multiple classification models intend to assess the net influences of age, period, or cohort on the outcomes of interest [3,11-16]. Age effects (A) represent the variation associated with different age groups brought about by biological, physiological, and behavioral changes, accumulation of social experience, and the role of status changes and events associated with growing up and aging [6,7,17]. Aging may lead to a decline in physical ability and functional capacity, such as an accumulation of oral health problems like caries and periodontal disease [6,7], which may finally affect tooth retention and also the probability of the individual's being edentulous. Period effects (P) represent variations over time periods that affect all age groups simultaneously in a certain historical period of time, often resulting from changes in social, cultural, economical, technological, or physical environments [7,17], such as changes in oral health policies or changes in income affecting the individual's ability to afford dental care, and, most importantly, advancements in technology, leading to the wide availability of preventive agents and effective treatments. Cohort effects represent variation across different (birth) cohorts caused by different long-term formative experiences, such as historical differences in social, economic, and physical environments, advances in technology, and changes in government policies from earlier years [7,12,17,18].

For trends in oral health or behavior to be identified and interpreted, it is important that the net influences of age, period, or cohort be determined. For example, changes in the utilization of dental services over time may be related to increasing oral health problems due to aging. However, period factors, such as the increasing dentist/population ratio, might play an important and simultaneous role. Birth cohort factors, such as the introduction of free dental care during school years, might influence the behavior of individuals during youth and adulthood.

### Conventional Age, Period, and Cohort analysis

Conventional statistical approaches have focused on modeling data at the population level, with cohort tables (age-by-period tables) of the rates (especially vital rates), and have been based mainly on the log-linear model or a linear model for the log rates [18]. The datasets used in conventional APC analysis included primarily information on age and period, or, in addition, on gender. Other possible important variables such as socio-economic status were generally absent in the dataset. This phenomenon was attributed to the fact that the datasets adopted were basically vital statistics provided mostly by statistics departments or hospital authorities. In conventional APC analysis, the identification problem (sometimes also known as the 'identifiability problem') inherent in the linear dependencies among age, period, and cohort (Age = Period - Cohort) should be noted. This means that if the survey year (period) and people's year of birth (cohort) are both known, then the age of the birth cohort at the survey year (age) is unequivocally fixed. Thus, the resulting regression coefficient estimates are not unique and cannot be used for statistical inference [19].

Over the past 30 years, various approaches have been applied to resolve this identification problem. Among those approaches, constraints were suggested to be imposed on any one of the three APC variables without affecting the underlying theoretical framework [13]. For example, two or more age groups might be combined into one group. However, different choices of constraint could result in different estimated APC effects [20]. Therefore, it is important that the particular constraint chosen be supported by prior theoretical arguments or empirical information. Replacing the concepts of age, period, and cohort by their underlying concepts has also been suggested [21]. An example of this strategy is the use of an appropriate psychological test, instead of age in general, to represent intellectual development [22]. This strategy can resolve the identification problem and provide easier interpretation of the Age, Period, and Cohort effects, since the concepts in question can be measured directly instead of through a proxy variable. If one of the APC variables could be measured in terms of the underlying variable, the linear dependency among the APC variables would disappear. Many studies have already reviewed and compared different approaches [18,20,22,23], but in summary, there are as yet no standard procedures to address the identification problem.

### Age, Period, and Cohort analysis in dentistry

Although APC analysis has been used by epidemiologists to identify and interpret temporal changes in oral health characteristics or behaviors in dental research [3-10], they were mainly descriptive, in the form of tables or graphs [4-6,8,10]. For example, Holst and Schuller [6] and Ahacic and Thorslund [10] adopted a descriptive approach on oral health changes [24], while Schwarz [4] and Sanders *et al. *[8] adopted separate regressions by year and descriptive age-standardized data by year and cohort, respectively, to describe the APC effects on oral health behavior. Only a few studies, like Bravo [7], followed the strategy proposed by Clayton and Schifflers [20,25] to analyze APC effects on the utilization of dental service over 10 years at the population level. However, Bravo's study [7] revealed a further risk of conventional APC analysis at the population level: that many other factors associated with dental demand were ignored in the analysis (such as school dental care during childhood, socio-economic status, denture-wearing, etc.).

### Suggested Age, Period, and Cohort analysis in sequential cross-sectional survey data

Ideally, longitudinal datasets should be collected and analyzed if APC analysis is to be applied at the individual level. In reality, however, very few longitudinal studies have been conducted for the purpose of APC analysis [26,27] in dental research. Instead, sequential cross-sectional data through repeated population surveys may have been collected and used for APC analysis [4,6,7]. In addition, cross-sectional sample survey research design yields other individual-level factors, besides age, period, and cohort, which are also associated with the variables of interest. This provides additional individual-level information for the development of alternative APC analysis. The challenge for APC analysis in sequential cross-sectional survey data is that conventional regression models have not taken into consideration the possibility that individuals are clustered in the same birth cohort surveyed at different survey years, and their responses or outcome variables may be similar because random errors unique to each cohort are common to each survey respondent in those cohorts [17,28]. Therefore, while the conventional regression models assume that the responses are independent, the results from these analyses without consideration of the possible clustering effect of birth cohorts may not be valid. Yang and Land [17,28] developed methodologies of hierarchical Age-Period-Cohort models for sequential cross-sectional surveys, and Yang [29] also applied that methodology to a dataset collected in the United States. In this project, a marginal approach was proposed for the analysis of sequential cross-sectional survey data by generalized estimating equations (GEE), to consider the possible clustering effect of birth cohorts.

It seems reasonable to provide here a brief description of the dental program for Danish children and the motivation for the choice of dental care during childhood as a proxy for the cohort effect in this analysis. Dental care for children in Denmark developed incrementally during the first part of the 19^th ^century, reaching mostly children in bigger cities or more affluent communities. A thorough historic account of this development has been provided by Lind *et al. *[30], and the organizational context of the children's dental services as a mandatory responsibility of the municipalities has been described by Kaplis *et al. *[31]. The oral health care system for children and adolescents was mandated by law by the Danish Parliament from 1972, and with amendments introduced in 1977, all children from birth to 18 years of age were offered systematic oral health care free of charge, comprised of general dental health promotion, individual prophylaxis, regular clinical examinations, and treatment. Because of the incremental system of the introduction of organized school dental care, the four study populations used in this analysis represent different birth cohorts with differential school dental care access. In 1975, the older age groups were unable to benefit from the dental care system, in contrast to the younger age groups. Decade by decade, as the school dental care system expanded to cover increasing proportions of the population, more people had the opportunity to benefit from the school dental care program. Thus, school dental care during childhood can be perceived as a proxy for the cohort effect and has been used in this analysis to resolve the identification problem and provide easier interpretation of the Age, Period, and Cohort effects.

The objective of this study was to analyze the effects of Age, Period, and Cohort on the regular dental care behavior and prevalence of edentulism in adult Danes reported in sequential cross-sectional oral health surveys by the use of school dental care during childhood as a proxy for cohort effects and application of a marginal approach to consider the possible clustering effect of birth cohorts as well as the effects of individual-level explanatory variables by GEE.

## Methods

### Study populations

We analyzed data from 4330 respondents aged 15+ years in 9 birth cohorts. The data were collected in 4 sequential cross-sectional surveys of non-institutionalized Danes. The four surveys were conducted in Denmark in 1975, 1985, 1995, and 2005. Each survey used a multi-stage stratified cluster sampling technique devised by the Statistical Bureau of Denmark (Danmarks Statistik) [4,32,33].

The sampling used for the surveys in 1975, 1985, and 1995 consisted of a methodology that drilled down through geographic areas until a specific address was reached according to an algorithm that would ensure statistical probability of representativeness at the national level. The sampling stages for the surveys in 1975, 1985, and 1995 consisted of random selection of 230 defined geographic districts from the whole country, mainly according to administrative divisions, stratification according to geographic (urban-rural) and occupational structure, selection of clusters of addresses within each geographic unit, random selection of clusters of households according to the area, and, finally, random selection of persons aged 15+ years from the selected households. (All members of selected households were listed in a fixed order, and every second person was randomly selected for questioning.) [4,32]. Up to three repeated visits were made to addressees who were not available on the first visit.

For the survey in 2005, a telephone survey was used. The sampling for the comparable telephone survey was similar in terms of ensuring geographic representation, but began with a national database of telephone numbers, which was 'cleaned' for mobile numbers and businesses. Each number was also attached to a geographic district coding, based on postal districts in the three major cities and on municipalities in the rest of the country. The selection of a nationally representative sample was achieved through a multi-stage process involving 8 geographic areas of the country, then the 16 counties, then the 276 municipalities, and finally 306 districts. Distribution of the sample took into account the proportional size of the population, which determined the number of respondents to be expected in a specific part of the country, corrected for extreme size, so that even small communities had a chance of being selected. Up to 7 repeat calls were made to addressees who were not available on the first call. Ultimately, the respondent database was established.

Both selection processes ensured a national probability sample of persons aged 15+ years. No weighting for failure cases was done. The sample sizes ranged from about 1000 to 1200 across the survey years. This corresponded to a response rate in each of the surveys of 71% (1995) to 80% (1975), with 1985 and 2005 situated between these. With the study populations in excess of 1000 respondents in each survey, the confidence intervals were narrow and of identical size. In each of the surveys, a core of identical questions was used on the basis of a structured questionnaire developed by one of the authors (ES) and carried out by a professional polling opinion research institute (Gallup Markedsanalyse A/S) in the spring of the four survey years [4,32].

### Study variables

The key study variables were seeking dental care on an annual basis over the preceding five years (ADC, regularly at least once a year in the preceding five years vs. not regularly every year) and edentulism (yes vs. no). The independent variables considered were: age (15-24, 25-34, 45-54, 55-64, 65-74, 75+ vs. 35-44); survey year (1975, 1985, 1995 vs. 2005); gender (male vs. female); socio-economic status (SES) group (low, medium vs. high), based on a composite socio-demographic variable recoded from original variables of occupation, income, and education; denture-wearing (both upper and lower dentures, either upper or lower denture only vs. no denture); and school dental care (SDC) during childhood (in all grades vs. not in all grades). In total, there were 4330 people aged 15+ years in the dataset. After the missing data were excluded (3.6%), there were 4172 individuals for the analysis of seeking ADC. For the analysis of edentulism, only respondents aged 35+ years were included. Since people younger than 35 years had a low probability of being edentulous, this age group was excluded from the analysis. After the missing data were excluded (4.9%), there were 2505 individuals for the analysis of edentulism.

### Statistical analysis

The distribution of respondents' demographic and other explanatory variables by year are summarized in Table 1 with the valid proportions. The proportion of respondents reporting seeking ADC and edentulism by year is also reported, with the overall proportion per age group listed next to the corresponding years in the figures. The effects of the selected explanatory variables on edentulism and ADC were analyzed by logistic regressions, with an independent correlation structure in GEE with the use of PROC GENMOD with the REPEATED statement in SAS software.

**Table 1:** The distribution and valid percentages of respondents according to demographics and other related oral health variables in 1975, 1985, 1995, and 2005.

	1975 (n = 1204)	1985 (n = 1123)	1995 (n = 1002)	2005 (n = 1001)
	**n (valid %)**	**n (valid %)**	**n (valid %)**	**n (valid %)**

**Age (yrs)**				
15-24	206 (17.1%)	188 (16.7%)	153 (15.3%)	118 (11.8%)
25-34	245 (20.3%)	206 (18.3%)	202 (20.2%)	159 (15.9%)
35-44	206 (17.1%)	251 (22.4%)	157 (15.7%)	212 (21.2%)
45-54	187 (15.5%)	136 (12.1%)	147 (14.7%)	181 (18.1%)
55-64	169 (14.0%)	144 (12.8%)	113 (11.3%)	170 (17.0%)
65-74	191 (15.9%)	120 (10.7%)	123 (12.3%)	103 (10.3%)
75+	/	78 (6.9%)	107 (10.7%)	58 (5.8%)
**Gender**				
Male	554 (46.0%)	515 (45.9%)	473 (47.2%)	427 (42.7%)
Female	650 (54.0%)	608 (54.1%)	529 (52.8%)	574 (57.3%)
**Socio-economic status (SES)**				
Low	413 (34.3%)	437 (38.9%)	385 (38.4%)	364 (36.4%)
Medium	676 (56.1%)	525 (46.7%)	460 (45.9%)	440 (44.0%)
High	115 (9.6%)	161 (14.3%)	157 (15.7%)	197 (19.7%)
**Denture-wearing**				
Both upper and lower dentures	298 (25.3%)	232 (20.7%)	181 (18.1%)	61 (6.1%)
Only upper or lower denture	149 (12.6%)	104 (9.3%)	82 (8.2%)	110 (11.0%)
No dentures	732 (62.1%)	787 (70.1%)	739 (73.8%)	830 (82.9%)
Missing	25 (-)	0 (-)	0 (-)	0 (-)
**School dental care (SDC) during childhood**				
At all grade levels	427 (36.3%)	703 (63.4%)	727 (72.6%)	867 (86.6%)
Not at all grade levels	750 (63.7%)	406 (36.6%)	275 (27.4%)	134 (13.4%)
Missing	27 (-)	14 (-)	0 (-)	0 (-)
**Seeking dental care on an annual basis during the preceding five years (ADC)**				
Regularly at least once a year	683 (58.8%)	780 (69.5%)	759 (75.7%)	823 (86.7%)
Not regularly every year	479 (41.2%)	343 (30.5%)	243 (24.3%)	126 (13.3%)
Missing	42 (-)	0 (-)	0 (-)	52 (-)
**Edentulism (age 35+ yrs)**				
Yes	269 (36.4%)	188 (26.3%)	133 (20.6%)	36 (5.0%)
No	471 (63.6%)	527 (73.7%)	514 (79.4%)	688 (95.0%)
Missing	13 (-)	14 (-)	0 (-)	0 (-)

Note that in 1975, the 75+ age group was included in the 65+ age group.

### GEE analysis

Marginal models such as GEE are appropriate when the interest of the study is not the clustering effect and their variances, but the inferences about the average response over the population and when the differences among clusters are minimal [34]. GEE was proposed for correlated data by Liang and Zeger [35,36], using the *quasi*-likelihood approach [37]. The GEE approach, extending the idea of the generalized linear model (GLM), assumes a known function of the marginal expectation of the dependent variables [38]. Liang and Zeger [36] proposed specifying the "working" correlation matrix for the observations among respondents from the same cluster to yield consistent estimators of the regression coefficients and their robust standard errors asymptotically, even when the "working correlation" structure is incorrect [35,36,38-40]. In consequence, robust standard errors are usually preferred. Unlike the ordinary regression analysis technique, the GEE allows one to account for possible correlation of responses from people within the same birth cohort.

From the logistic regressions performed for each survey year separately, it was observed that there were homogeneous effects in some age groups (not reported here). Therefore, for the analysis of seeking ADC in this project, age would be regrouped as 15-24, 25-34, 35-44, 45-64, and 65+ years, with the 35- to 44-year age group as the reference category. And for the analysis of being edentulous, age would be regrouped as 35-44, 45-64, 65-74, and 75+ years, with the 35- to 44-year age group as the reference category.

In addition, the possible clustering effect of birth cohorts was considered in the GEE models, but the cohort effect was not explicitly estimated. SDC during childhood was used as the proxy of the cohort effect. This strategy resolved the identification problem and provided easier interpretation of the cohort effect.

For the GEE analysis of ADC, the explanatory variables were age (15-24, 25-34, 45-64, 65+ vs. 35-44), survey year, gender, SES group, denture-wearing, and SDC during childhood. For the GEE analysis of edentulism, only respondents aged 35+ years were included. The explanatory variables were age (45-64, 65-74, 75+ vs. 35-44), survey year, gender, SES group, ADC, and SDC during childhood.

Because the GEE model is not estimated by full-information maximum likelihood, the widely used tests such as the likelihood ratio test, Akaike's Information Criterion (AIC), and Bayesian Information Criteria (BIC) for model fit, penalized model selection, and block significance testing may not be appropriate. Fortunately, the *Quasi*-likelihood under the Independence model Criterion (QIC) statistic proposed by Pan [41] is analogous to the AIC statistic and can be used to compare GEE models for the selection of regression models and working correlations. The model with a smaller QIC is more preferable, and most statistical packages (e.g., SAS) that implement GEE also provide procedures for conducting such tests.

In this project, the QIC statistic was used for GEE model selection. The GEE results were expressed as odds ratios and corresponding 95% confidence intervals (CI), and the associated p-value of Wald's test for test of significance were also reported. All statistical tests were performed with two-sided tests at the 0.05 level of significance. All analyses were performed with SAS version 9.2 (Cary, NC, USA).

## Results

### Overall description

In 1975, the 75+ age group was included in the 65+ age group. In subsequent surveys, this distinction was recorded into age groups 65-74 and 75+. Table 1 reports the distribution of demographics and other related oral health variables (age, gender, SES, denture-wearing, and SDC during childhood) by year. The numbers of non-missing observations in the corresponding variable are also listed. The distributions of age were generally bell-shaped (Table 1), while gender was almost evenly distributed. Nearly half of those in the socio-economic status group were in the medium category, with 56.1% in 1975 slightly decreasing to 44.0% in 2005. In 1975, 62.1% of the respondents were not wearing dentures, and this proportion increased to 82.9% in 2005. The percentage of the respondents receiving SDC in all grades during childhood increased from 36.3% in 1975 to 86.6% in 2005.

### Seeking dental care on an annual basis (ADC)

#### Descriptive results

**Figure 1:**
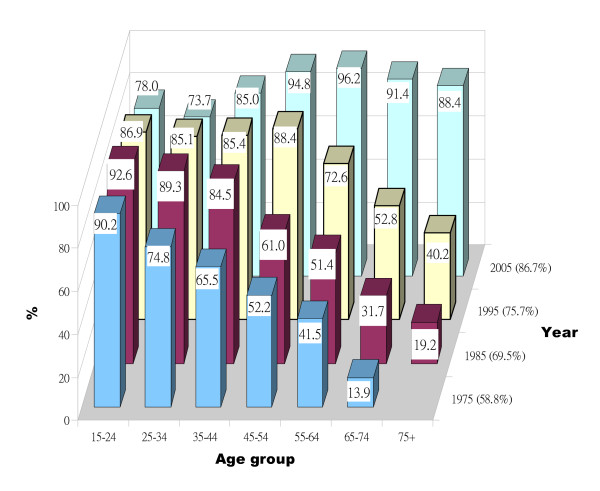
**Regular dental care behavior among adult Danes**. Proportion of respondents by age group who reported seeking dental care on an annual basis during the five years preceding the survey year. Note: in 1975, the 75+ age group was included in the 65+ age group.

Corresponding to the incremental growth of the children's oral health care services, only 36% of the 1975 population reported having had dental care at all grades during their school years, with considerable variation between the age groups (3% of the oldest age group compared with 70% of the youngest). This proportion increased dramatically during each decade, to 63% in 1985, 73% in 1995, and 87% in 2005. In the overall population, the proportion of people seeking ADC increased from 58.8% (95% CI, 56.0%-61.6%) in 1975 up to 86.7% (95% CI, 84.5%-88.9%) in 2005 (see Table 1). Temporal changes in the proportions of people seeking ADC were observed for all age groups from 1975 to 2005 (Figure 1). The percentage of people seeking ADC generally decreased with increasing age, but over the survey years, those in the older age group tended to maintain the high dental care utilization rate from their youth (for instance, the 15- to 24-year-olds in 1975, who were 45-54 in 2005), whereas the younger age groups in the later surveys were less prone to report regular dental care (for instance, 15- to 24- and 25- to 34-year-olds in 2005).

#### GEE results

Based on the QIC statistic for GEE model selection, the explanatory variables of the final model were age, survey year, gender, SES group, denture-wearing, SDC during childhood, and the interaction between age and SDC during childhood (*P *< 0.05) (Table 2). The females were associated with a higher probability of seeking ADC regularly, as shown in the result that the odds of "seeking ADC regularly" was lower in males than in females [Odds Ratio (OR) = 0.56; 95% CI, 0.50-0.62; *P *< 0.0001]. Similarly, when compared with the high-SES group, the odds of "seeking ADC regularly" in the low- and medium-SES groups were significantly smaller, with respective OR 0.39 (95% CI, 0.27-0.55) and 0.58 (95% CI, 0.39-0.86). Respondents not wearing any denture were much more likely to seek ADC regularly than were respondents with both upper and lower dentures (OR = 0.06; 95% CI, 0.05-0.08) and those with only either an upper or a lower denture (OR = 0.41; 95% CI, 0.29-0.58).

**Table 2:** Odds ratios with 95% confidence intervals (CI) and P-values of Wald's tests of the final model for the probability of seeking ADC regularly by Danes aged 15 - 75+ in Denmark, 1975 - 2005.

Explanatory Variable	Odds ratio	(95% CI)	P-value
**Age (yrs)**			0.1491
65+			
45-64			
35-44^a^			
25-34			
15-24			
			
**Survey Year**			< 0.0001*
1975	0.44	(0.19, 0.97)	
1985	0.70	(0.31, 1.59)	
1995	0.94	(0.52, 1.71)	
2005^a^		1	
			
**Gender**			< 0.0001*
Male	0.56	(0.50, 0.62)	
Female^a^		1	
			
**Socio-economic status (SES)**			< 0.0001*
Low	0.39	(0.27, 0.55)	
Medium	0.58	(0.39, 0.86)	
High^a^		1	
			
**Denture-wearing**			< 0.0001*
Both upper and lower	0.06	(0.05, 0.08)	
Only upper or lower	0.41	(0.29, 0.58)	
No denture^a ^		1	
			
**School dental care (SDC) during childhood**			
In all grades			0.0193*
Not in all grades^a^			
			
Age * SDC during childhood			< 0.0001*
65+ and in all grades	1.70	(0.40, 7.31)	
65+ and not in all grades	0.60	(0.31, 1.15)	
45-64 and in all grades	1.69	(0.76, 3.76)	
45-64 and not in all grades	0.74	(0.54, 1.02)	
35-44 and in all grades	0.91	(0.66, 1.24)	
35-44 and not in all grades^a^		1	
25-34 and in all grades	0.87	(0.28, 2.70)	
25-34 and not in all grades	0.76	(0.46, 1.24)	
15-24 and in all grades	1.58	(0.10, 25.53)	
15-24 and not in all grades	1.50	(0.48, 4.66)	
			

^a ^Reference group; * Significant result, *P *< 0.05

Note that only the main effects of age and SDC during childhood are shown with their interaction effect in the Table.

**Figure 2:**
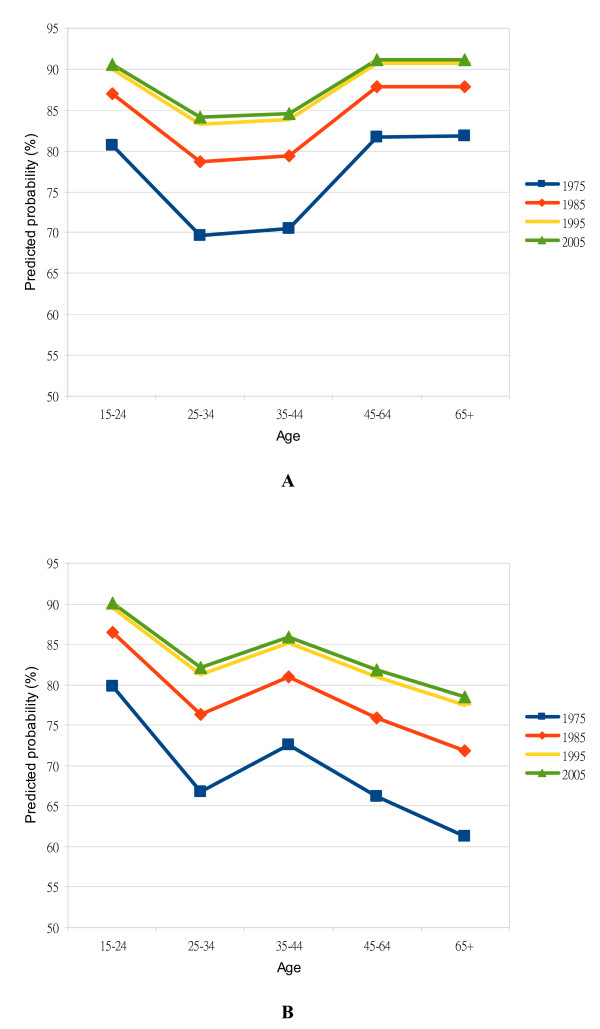
**Corresponding probabilities of seeking annual dental care (ADC) among males - school dental care (SDC) in and not in all grades respectively. **Figure A: Predicted probability of seeking ADC regularly for males in the medium-SES group with no denture and SDC in all grades during childhood in relation to age group and survey year. Figure B: Predicted probability of seeking ADC regularly for males in the medium-SES group with no denture and SDC not in all grades during childhood in relation to age group and survey year.

The interaction effect of age and SDC during childhood was highly significant (P < 0.0001). To summarize, people who received SDC in all grades during childhood, especially the elderly, had a higher probability of seeking ADC. As an illustration, Figures 2A and 2B contrast the predicted probabilities of seeking ADC regularly in those males in the medium-SES group with no denture who had SDC in all grades during childhood and those males who had not had SDC in all grades. People receiving SDC in all grades during childhood, especially the elderly, had increased probability of seeking ADC. The predicted probabilities for those receiving SDC in all grades during childhood were maintained at around 70% to 90% (Figure 2A). In contrast, the predicted probabilities for those not receiving SDC in all grades during childhood were generally lower across all age groups, with a decreasing trend after 44 years of age (Figure 2B).

Also, it is apparent that the period effect increased from 1975 to 2005, while the increase from 1995 to 2005 was greatly narrowed (Figures 2A and 2B). This can also be shown in Table 2, in which the adjusted odds ratios of seeking ADC regularly in the respondents surveyed in the survey years 1975, 1985, and 1995 compared with the survey year 2005 were 0.44 (95% CI, 0.19-0.97), 0.70 (95% CI, 0.31-1.59), and 0.94 (95% CI, 0.52-1.70), respectively, adjusted for other explanatory variables. These all showed that the respondents surveyed in the recent survey years were associated with a higher probability of seeking ADC regularly (*P *< 0.0001).

### Edentulism

#### Descriptive results

**Figure 3:**
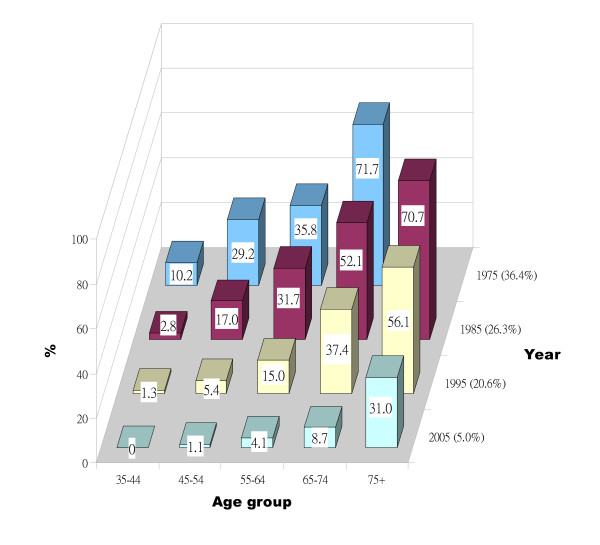
**Edentulism among adult Danes**. Proportion of respondents who reported to be edentulous in relation to age group and survey year. Note: the z-axis (survey year) is the reverse of that in Figure 1 and in 1975, the 75+ age group was included in the 65+ age group.

In the population aged 35 years old or above, the prevalence of edentulism decreased from 36.4% (95% CI, 32.9%-39.9%) in 1975 to 5.0% (95% CI, 3.4%-6.6%) in 2005 (see Table 1). Temporal declines in the prevalence were reported for all age groups from 1975 to 2005 (Figure 3), with the greatest absolute decrease observed between 1995 and 2005, especially for the elderly (the 65+ age groups). Prevalence of edentulism for the 65-74 age group and 75+ age group dropped from 37.4% and 56.1% in 1995 to 8.7% and 31.0% in 2005, respectively. Overall prevalence of edentulism for the elderly (the 65+ age groups) had a remarkable drop, from 46.1% in 1995 to 16.8% in 2005.

#### GEE results

Based on the QIC statistic for GEE model selections, the explanatory variables of the final model were age, survey year, gender, SES group, ADC, and SDC during childhood. All the factors were significant in the logistic regression by GEE, with an independence working correlation matrix (*P *< 0.05) (Table 3). Males were less susceptible to "being edentulous" than were females (OR = 0.42; 95% CI, 0.29-0.61; *P *< 0.0001). The odds of "being edentulous" was highest among respondents in the low-SES group and lowest among those in the high-SES groups [odds ratios of the low- and medium-SES groups over the high-SES group were, respectively, 5.18 (95% CI, 3.19-8.43) and 2.03 (95% CI, 1.20-3.43)].

**Table 3:** Odds ratios with 95% confidence intervals (CI) and P-values of Wald's tests of the final model for the probability of being edentulous of Danes aged 35 *- *75+ in Denmark, 1975 *- *2005.

Explanatory Variable	Odds ratio	(95% CI)	P-value
**Age (yrs)**			< 0.0001*
75+	13.80	(9.60, 19.85)	
65-74	6.75	(4.86, 9.37)	
45-64	3.60	(2.52, 5.16)	
35-44^a^		1	
			
**Survey Year**			< 0.0001*
1975	11.48	(7.77, 16.98)	
1985	7.92	(5.15, 12.18)	
1995	7.10	(5.03, 10.02)	
2005^a^		1	
			
**Gender**			< 0.0001*
Male	0.42	(0.29, 0.61)	
Female^a^		1	
			
**Socio-economic status (SES)**			< 0.0001*
Low	5.18	(3.19, 8.43)	
Medium	2.03	(1.20, 3.43)	
High^a^		1	
			
**ADC**			< 0.0001*
Regular	0.03	(0.02, 0.05)	
Not regular^a^		1	
			
**School dental care (SDC) during childhood**			0.0386*
In all grades	0.60	(0.37, 0.97)	
Not in all grades^a^		1	

^a ^Reference group; * Significant result, *P *< 0.05.

**Figure 4:**
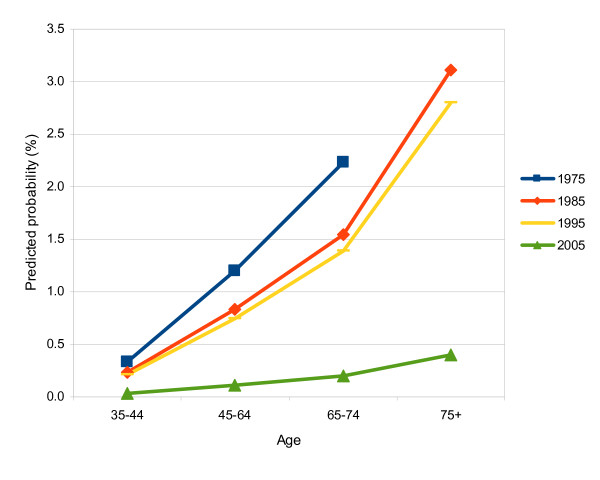
**Probability of edentulism among males who sought regular dental care**. Predicted probability of being edentulous among males in the medium-SES group in relation to age group and survey year. These respondents sought annual dental care regularly and had received school dental care in all grades during childhood.

**Figure 5:**
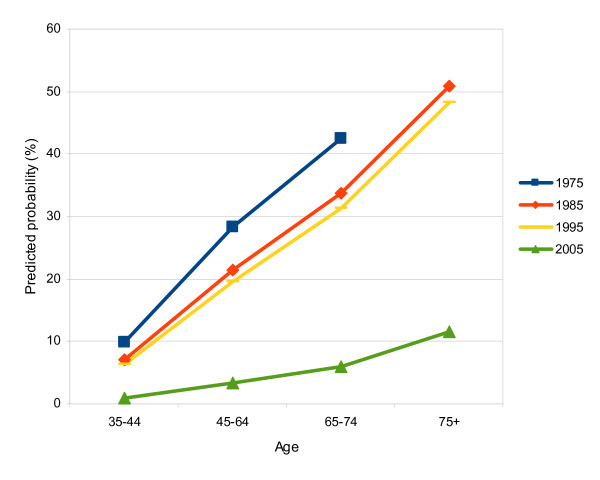
**Probability of edentulism among males who did not seek regular dental care**. Predicted probability of being edentulous among males in the medium-SES group in relation to age group and survey year. These respondents did not seek annual dental care regularly and had received school dental care in all grades during childhood.

The predicted probabilities of being edentulous for males in the medium-SES group who had received SDC in all grades during childhood and who sought ADC regularly compared with those males who did not seek ADC regularly are displayed in Figure 4 and 5 to explain the effects more clearly. The age effect (*P *< 0.0001, Table 3) was very apparent, with the predicted probability of being edentulous for respondents in the older age group being several-fold that of respondents in the younger age group. The adjusted odds ratios of "being edentulous" in the 75+, 65-74, and 45-64 age groups compared with the 35-44 age group were 13.80 (95% CI, 9.60-19.85), 6.75 (95% CI, 4.86-9.37), and 3.60 (95% CI, 2.52-5.16), respectively (Table 3).

The period effect can also be demonstrated in Figures 4 and 5. The predicted probabilities sharply decreased from 1975 to 1985, then slightly decreased from 1985 to 1995, and finally dropped substantially from 1995 to 2005. This can also be shown in Table 3, in which the period effect was significant with *P *< 0.0001, and the adjusted odds ratios in the respondents surveyed in the survey years 1975, 1985, and 1995 compared with the survey year 2005 were 11.48 (95% CI, 7.77-16.98), 7.92 (95% CI, 5.15-12.18), and 7.10 (95% CI, 5.03-10.02), respectively.

Besides, respondents who had received SDC in all grades during childhood were significantly less susceptible to "being edentulous" (OR = 0.60; 95% CI, 0.37-0.97; *P *= 0.0386) (Table 3).

In contrast, respondents seeking ADC regularly were significantly less susceptible to "being edentulous" (OR = 0.03; 95% CI, 0.02-0.05; *P *< 0.0001). The predicted probabilities for those seeking ADC regularly ranged only from 0 to 3.5% (Figure 4). In contrast, the predicted probabilities for those who did not seek ADC regularly had a surprisingly sharp increase of around 10 times the probability of edentulism of the former (Figure 5).

## Discussion

### Topical studies in Denmark and internationally

The present study provided us with the opportunity to review the changes in edentulism and regular dental visit habits in adult Danes over a long time period, and to identify the potential factors that have affected the two developments, such as attending school dental care during childhood in addition to the effects of age, period, and cohort over the past three decades. Thus, the study brings together a range of reports which have addressed the oral health status and demand for dental care in adult Danes at a single point in time during this period [4,32,42-54]. The findings in the present study show a marked improvement in the quality of dental health service and dental visiting habits of adults in Denmark. The results show that absence from annual dental visits and edentulism were associated with the low socio-economic status of the respondents, in agreement with recent reports on social inequities in oral health status and use of dental health services among adult Danes [42-44]. The increase in regular dental visits was significantly related to whether the individuals wore any denture. Similar trends have been found in other Western countries, such as Australia [55], the United Kingdom [56], Sweden [10], and the USA [57,58]. The differences observed in regular dental visiting habits in terms of gender were consistent with worldwide trends and have been reported consistently over the past 10 years [42,59]. The results demonstrate the successful long-term effects of public oral health policy for children and adolescents in Denmark. Respondents who attended school dental care during all grades during childhood had significantly less probability of being edentulous as adults and had significantly higher probability of seeking regular ADC.

The findings confirm previous reports regarding the positive effect of regular dental care during childhood on dentate status in adulthood [43]. Moreover, the present study indicates that the risk of being edentulous still remains large if the respondents do not develop the good habit of annual dental visits, even though they attended school dental care in all grades. Therefore, the oral health care system for children and adolescents in Denmark reduced the number of people being edentulous mainly through education and providing regular school dental care during childhood to build a habit of annual dental visits, but not directly through providing school dental care during childhood *per se*.

### Applications of GEE

Generalized estimating equations have been applied in dental studies for decades. They have been used to account for correlated observations in different dental fields, such as periodontology [60,61], implant dentistry [62,63], endodontics [64], and caries research [65].

In this project, the interests focus on how the response variables are affected by the explanatory variables in the presence of some potential clustering cohort effects. To consider the possible clustering effect of birth cohorts, we applied a marginal approach to sequential cross-sectional survey data by means of generalized estimating equations (GEE). The marginal approach is referred to as a "population-averaged" approach, which makes statistical inferences about the average response over the population, while a random-effects approach is referred to as a "subject-specific" approach to model the effect of changing one or more explanatory variables on a given individual. The studies by Yang and Land [17,28] are some examples applying the random-effects approach. The comparison of these two approaches has been discussed in some of the statistical and epidemiological literature [39,40,66-72]. The main critical consideration for choosing the appropriate approach to data analysis depends on whether the magnitude of the clustering effect of correlated responses is of interest. Therefore, if the interest focuses on how the individual response over the population is affected by the clustering effect, the random-effects approach is recommended, providing an alternative approach to accommodate correlated responses. Otherwise, the marginal approach is more appropriate because of its simplicity.

### Age effects on seeking ADC and being edentulous

The age effect on both seeking ADC and edentulism was significant when adjusted for other explanatory variables and when the potential clustering effect of birth cohorts was considered. When adjusted for gender, SES, ADC, and SDC during childhood, the age effect on seeking ADC was reflected in the significant interaction effect between age and SDC during childhood in the extended APC model. In general, the effect of age first dropped from adolescence (15-24 years) to early adulthood (25-34), and was then maintained or increased slightly again in the later years (35-44 years). This might be because if the adolescents received dental care at school in all grades, they would naturally have a high probability to seek ADC. However, perhaps due to the high maintenance of oral health status during childhood and adolescence, the young adults (25-44 years) had no urgent need to seek ADC immediately. But then later, due to increasing oral health problems related to aging, more people aged 65+ years began to seek ADC. But then the effect of age in middle age and late adulthood was shown to depend greatly on SDC during childhood.

Similarly, the findings in this paper revealed that the age effect was significant in the extended APC model of being edentulous after adjustment for other explanatory variables. In general, the findings revealed that adults had a higher risk of being edentulous due to aging. This finding confirmed the results of a previous study on edentulism [73].

### Period effects on seeking ADC and being edentulous

The period effect on both seeking ADC and edentulism was also significant when adjusted for other explanatory variables and when the potential clustering effect of birth cohorts was considered. On one hand, the findings demonstrated that the period effect was significant in the extended APC analysis of seeking ADC. The period effect on seeking ADC increased gradually from the survey years 1975 to 2005. This might be due to more dental education in advertising media and more widespread availability of dental service providers, which created convenience for people to develop the habit of seeking ADC.

On the other hand, larger odds ratios of the earlier survey years (such as the value 11.48 for the survey year 1975 compared with the survey year 2005) in the model of edentulism suggested a dramatic reduction in the prevalence of edentulousness as a great improvement of oral health status in Denmark in recent years. Danes in the more recent survey years had a lower risk of being edentulous. This might be due to the technological advancement in dentistry, the improvement in the quality of dental service, and more widespread availability of dental service providers, increasing convenience for people seeking to visit dentists when they suffered from oral health problems. But the difference in period effect was comparatively small between the survey years 1995 and 2005. This could be interpreted as the recent maturity of the advancements in dental technology and the practice of dentistry.

### Effect of school dental care during childhood

In this paper, SDC during childhood was used as a proxy for cohort effects. Cohort effects on seeking ADC and being edentulous can be partially reflected by the significant effect of SDC during childhood.

First, from the model of seeking ADC, among the respondents who had received SDC in all grades during childhood, the probability of seeking ADC increased continuously in the later stage of life. Otherwise, they had a continuously decreased probability of seeking ADC from middle age (45+ years). These findings suggested that those cohorts who enjoyed the benefits from the dental care system (that is, who had received SDC in all grades during childhood) were relatively successful in maintaining the probability of seeking ADC throughout their lives.

Second, SDC during childhood also had a significant effect in the model of edentulism. Among the respondents who had received SDC in all grades during childhood, their risk of being edentulous was reduced by 40%, compared with those who had not. These findings suggested that those cohorts who enjoyed the benefits from the dental care system (that is, who had received SDC in all grades during childhood) had an apparently reduced risk of being edentulous compared with the earlier cohorts.

### Limitations

Threats to the external validity of these studies refer especially to the size of the non-response of the original sample and to the composition of the resulting study populations. Due to the extensive experience of the polling institute responsible for the data collection in drawing the national sample, and the compensatory mechanisms developed to reduce bias, such as repeated visits/calls and replacement methodologies, the populations under study maintained a close similarity to the Danish population sampling frame in terms of age and gender. Since the interview methodology underwent a change in 2005, which was beyond the influence of the researchers, it is possible that a slightly different pattern in reporting of health behavior and oral health status was introduced. No systematic differences could be observed in any of the comparable demographic variables. Slight over-reporting on the habit of regular dental visit or under-reporting on edentulism might be expected. However, as noted in a previous study [74], there is little evidence of a general tendency for telephone respondents to report more health events than respondents interviewed in person. Studies of the demand for dental visits are based mainly on self-reporting, and this method is considered valid, although some over-reporting may occur [75]. Also, in interview surveys, recall bias regarding school dental care during childhood is possible, particularly among older adult age groups.

## Conclusions

Failure to account appropriately for the potential clustering effect of birth cohorts in the analysis of data from sequential cross-sectional studies may lead to misrepresentation of the data or, worse, to invalid statistical inferences on the significance of certain factors. With the use of GEE, which is easy to implement and gives efficient estimates for the regression coefficients under weak correlation assumptions, the clustering effect of birth cohorts that may exist in sequential cross-sectional oral health survey data could be appropriately accounted for in the inferential procedures. Thus, we are confident in concluding that the dental health policy in Denmark was successful in a continued increase of regular dental visiting habits and tooth retention in adults by providing school dental care to Danes in their childhood.

## Competing interests

The authors declare that they have no competing interests.

## Authors' contributions

ES conceived of and carried out the surveys in Denmark during 1975-2005. As part of her MPhil studies, KYL conducted all the statistical analyses under the supervision of MCMW and KFL. All authors contributed to the preparation of the manuscript. All authors have read and approved the final version of the manuscript.

## Pre-publication history

The pre-publication history for this paper can be accessed here:

http://www.biomedcentral.com/1472-6831/11/9/prepub
